# Cellulose nanofiber reinforced curcumin-infused calcium phosphate silicate cement for various bone-tissue engineering application

**DOI:** 10.3389/fonc.2024.1516638

**Published:** 2025-01-14

**Authors:** Xiu Guo Lu, Sha Li Meng, Qiu Jing Zhou, Tao Wu, Xing Tian Gong, Qiong Wu

**Affiliations:** ^1^ College of Information Science and Engineering, Northeastern University, Shenyang, Liaoning, China; ^2^ Department of Nuclear Medicine, General Hospital of Northern Theater Command, Shenyang, Liaoning, China; ^3^ Department of Biomedical Engineering, Shenyang University of Technology, Shenyang, China; ^4^ Department of Neurology, Shengjing Hospital of China Medical University, Shenyang, Liaoning, China

**Keywords:** curcumin, calcium phosphate silicate cement, bone cancer, bone repair, dual functions

## Abstract

**Introduction:**

This study utilized a injectable curcumin (Cur)-infused calcium phosphate silicate cement (CPSC) for addressing defects caused by bone cancer, and evaluated its promoting bone regeneration and exerting cytotoxic effects on osteosarcoma cells.

**Methods:**

The material’s physicochemical properties, biocompatibility with osteoblasts, and cytotoxicity toward osteosarcoma cells were rigorously analyzed.

**Results:**

The findings demonstrate that CPSC-Cur signicantly prolongs the setting time, which can be optimized by adding silanized cellulose nanober (CNF-SH) to achieve a balance between workability and mechanical strength. Biological assessments reveal a pronounced cytotoxic effect on osteosarcoma cells while maintaining minimal toxicity toward pre-osteoblasts, highlighting CPSC-Cur’s potential as a promising material for repairing bone defects following cancer removal.

**Conclusion:**

This study lays the groundwork for future investigations into CPSC-Cur’s *in vivo* efficacy and its role in the clinical treatment of bone cancer.

## 1 Introduction

Bone tissue engineering stands at the frontier of regenerative medicine, presenting promising avenues for the repair of damaged or defective bone tissues through the application of advanced biomaterials as suitable scaffolds (1). The challenge becomes markedly more complex in the realm of bone cancer, where biomaterials must not only support new bone growth but also simultaneously counteract malignant cells. This dual challenge stems not only from the lethal nature of cancer but also from its profound impact on bone regeneration and structural integrity (2). Both primary bone cancers, such as osteosarcoma, and secondary metastatic cancers, which originate in other organs and metastasize to the bone—like breast cancer—disrupt the delicate equilibrium of bone remodeling (3, 4). These malignancies can lead to an excessive production of osteoclasts or the formation of osteoblast-like cells that produce abnormal and structurally compromised bone tissue, rendering it more prone to fractures. Refractory bone defect typically refers to a bone defect that is difficult to heal or repair through conventional treatment methods due to its severity, size, or location. These defects can arise from various causes, including trauma, tumor resection, infection, or diseases like osteoporosis and ostenecrosis (5). Due to their challenging nature, these bone defects often require specialized treatment strategies, such as autologous or allogeneic bone grafting, biomaterial scaffolds, or advanced regenerative medical technologies to promote bone regeneration and repair (6).

Consequently, the pursuit of new biomaterials that can fulfill these dual roles is vigorous, underscoring a pivotal area of investigation within biomaterial research. Biomaterial strategies in the battle against bone cancer have undergone substantial evolution, emphasizing the utilization of sophisticated materials for the efficient delivery of antitumor drugs to afflicted areas. Prominent methods include employing adaptable biomaterials engineered for the precise conveyance of antitumor agents, such as 5-fluorouracil (5-FU), either singly or in synergy with other anticancer techniques like photothermal or magnetic thermal therapies, directly targeting the tumor (7–9). These approaches significantly bolster treatment effectiveness while reducing systemic adverse effects. Furthermore, within the sphere of nanotechnology, the advent of multifunctional bionanomaterials marks a noteworthy development, highlighting the innovative combination of drug delivery mechanisms with cancer-targeting features to yield a more concentrated and powerful attack on bone cancer (8, 10).

Despite the promising approach of using biomaterials impregnated with antitumor drugs to combat bone cancer, several obstacles and constraints diminish their efficacy. A primary challenge lies in achieving selective targeting of cancer cells while safeguarding healthy bone tissue, a critical equilibrium essential for preserving the integrity of healthy bone and reducing adverse effects associated with pharmacological and surgical treatments (11). Moreover, the high costs of producing nanomaterials, coupled with their low targeting precision and the potential toxicity due to abrupt drug release, further impede the successful treatment of bone cancer with drug-laden biomaterials (7, 12, 13). In recent years, curcumin (Cur), a naturally occurring polyphenolic compound prevalent in turmeric, has gained considerable recognition for its robust antitumor qualities (14). Numerous investigations have explored its antimicrobial, anti-inflammatory, anti-arthritic, and anti-cancer properties (15). Notably, several studies have elucidated curcumin’s role in modulating the proliferation and differentiation of bone cells, thereby enhancing bone formation (16). Its broad array of additional benefits, including anti-inflammatory and antioxidant properties, are attributed to its ability to influence a variety of molecular targets involved in cancer’s advancement, such as hindering cell growth, promoting apoptosis in tumor cells, and disrupting cancer cell communication. Furthermore, curcumin bolsters the effectiveness of standard chemotherapy agents like 5-fluorouracil, thereby serving as a valuable adjuvant in cancer treatment. Curcumin emerges as a promising anti-inflammatory agent in the management of glucocorticoid-induced osteonecrosis, concurrently alleviating osteocyte apoptosis within the femoral head (17). Through its multifunctional properties, curcumin emerges as a potential therapeutic agent for addressing various aspects of bone tissue homeostasis and pathology. Moreover, curcumin demonstrates the capacity to impede the proliferation, differentiation, and migration of osteoclasts, crucial in preventing excessive bone resorption and maintaining bone density (18).

In this present study, we have developed an innovative curcumin delivery system utilizing calcium phosphate silicate bone cement (CPSC). Previous research has demonstrated CPSC’s efficacy as a drug delivery platform and its significant role in promoting bone regeneration (19, 19). Furthermore, its injectable form offers remarkable versatility and ease of application, adapting seamlessly to various bone defect geometries (20, 21). This investigation aims to thoroughly assess the potential of curcumin-enriched CPSC (CPSC-Cur) as an advanced biomaterial for bone tissue engineering, especially in contexts complicated by bone cancer. Our objectives are to characterize the physicochemical properties of CPSC-Cur, evaluating its biocompatibility with osteoblasts, and investigate its cytotoxic effects on osteosarcoma cells. The importance of this research lies in its potential to contribute a novel biomaterial that not only promotes bone regeneration but also provides a strategic edge in combating bone cancer, thereby addressing a critical gap in the current treatment approaches.

## 2 Material and methods

### 2.1 Chemicals and reagents

Monocalcium phosphate (Ca(H_2_PO_4_)_2_, MCP), calcium nitrate tetrahydrate (Ca(NO_3_)_2_·4H_2_O), and tetraethyl orthosilicate (Si(OC_2_H_5_)_4_) were sourced from Sigma Aldrich, China. Curcumin (Cur) was acquired from Tianjin Heowns Biochemical Technology, China. L-glutamine, Alpha Minimum Essential Medium (α-MEM), phosphate-buffered saline (PBS), penicillin-streptomycin, and trypsin-EDTA were supplied by Invitrogen, China. McCoy’s 5A (Modified) Medium and fetal bovine serum (FBS) were obtained from Gibco, China. The Cell Counting Kit-8 (CCK-8) assay was purchased from Dojindo, China and Annexin V-APC/PI apoptosis detection kit was procured from KeyGen Biotech, China. Dimethyl sulfoxide (DMSO) and EDTA-free trypsin were sourced from Solarbio, China. Silanized cellulose nanofiber (CNF-SH, diameter = 30 – 50 nm, length = 0.8 – 1 μm) was acquired from Century Cellulose Company, Jiangsu, China.

### 2.2 Preparation of Cur-CPSC pastes

Dicalcium silicate (Ca_2_SiO_4_, C_2_S) and tricalcium silicate (Ca_3_SiO_5_, C_3_S) were synthesized via a sol-gel method as described previously. The CPSC composition comprised equal mass fractions of C_2_S and C_3_S with variable proportions of MCP. The CPSC powders were uniformly ground using a ball mill (YXQM-2L, MITR, China).

We initially investigated the incorporation methods of Cur into the CPSC, assessing its impact on material properties. CPSC powders with 15% MCP were blended with either 5% Cur powders and deionized water (ddH_2_O) simultaneously or with Cur-dissolved ddH_2_O (equivalent amount of Cur). The resulting pastes were labeled CUR@CS15P(S) and CUR@CS15P(L), respectively.

We further investigated the reinforcement of Cur-CPSC with CNF-SH by integrating various concentrations of CNF-SH directly into the CPSC powders. The formulations included 1%, 2%, and 3% CNF-SH mixed with CPSC powders, designated as 1CNF-CUR@CS15P(S), 2CNF-CUR@CS15P(S), and 3CNF-CUR@CS15P(S), respectively. Additionally, we prepared CNF-SH enriched formulations, in which Cur was blended with CPSC in the solution form, at concentrations of 1%, 1.5%, 2%, and 3%, labeled as 1CNF-CUR@CS15P(L), 1.5CNF-CUR@CS15P(L), 2CNF-CUR@CS15P(L), and 3CNF-CUR@CS15P(L), respectively. All these formulations consistently contained a 15% mass fraction of MCP. The preparation of all samples kept a 5% mass fraction of Cur.

Finally, we adjusted the MCP concentration to formulate a range of MCP-doped Cur-CPSC pastes, designated as CS, CS1P, CS2P, CS3P, CS4P, CS5P, CS10P, and CS15P, corresponding to MCP concentrations from 0% to 15%. In these variations, 5% Cur was dissolved in deionized water (ddH_2_O), and 1.5% CNF-SH was directly blended with the CPSC powders, following the methodology previously described. The preparation of all samples maintained a consistent liquid-to-powder ratio of 0.5 mL/g.

### 2.3 Material characterization of Cur-CPSC paste

#### 2.3.1 Setting time

The Cur-CPSC paste’s setting time was determined using a Vicat needle apparatus, following ASTM standard C191-18 with minor modifications. The paste was placed in a custom plastic mold (diameter = 17 mm, height = 2 mm) at 37°C and 100% relative humidity. Assessments were made at 5 to 10-minute intervals until the Vicat needle left no visible mark on the paste surface, indicating the final setting time. Each group had three replicates (n=3).

#### 2.3.2 Compressive strength

The compressive strength of the Cur-CPSC pastes was evaluated using a universal testing system (UTS, Instron 5944, U.S.A.). The paste was cast in a custom cylindrical mold (diameter = 6 mm, height = 12 mm) and hydrated at 37°C and 100% relative humidity. For the CS, CS1P, CS2P, CS3P, CS4P, CS5P, CS10P, and CS15P groups, the hardened samples were tested after 3 days using a 2 kN loading cell at a 1 mm/min crosshead speed. The CS5P group samples were also tested after 5 and 7 days. Each group had four replicates (n = 4).

#### 2.3.3 Crystallinity, chemical and microstructure analysis

Crystallinity was determined by X-ray diffraction (XRD, D/Max-3B, Rigaku, Japan) using JADE software (MDI, U.S.A.) and the ICDD PDF-4 database. Fourier transform infrared (FT-IR) spectra were recorded using an FT-IR spectrometer (Nicolet iS50, Thermo Fisher Scientific, U.S.A.) in the range of 3600 cm^-1^ to 400 cm^-1^ with a resolution of 4 cm^-1^.

#### 2.3.4 Anti-washout, injectability and cohesiveness

In the anti-washout ability test, 15 mm diameter balls were handcrafted using freshly prepared cement paste and sequentially placed in saline solution at 1-minute intervals, as previously established (21). The balls were considered washout-proof if no surface abrasion was visible after scrubbing them by hand in saline solution for 20 seconds. The elapsed times were recorded, with each group having three replicates (n = 3).

The paste injectability and cohesion were also measured according to our previous protocols. Briefly, the paste was filled into a 5 mL plastic syringe with a 2 mm orifice. The period between paste mixing and extrusion was kept under 2 minutes to minimize the initial setting effects on injectability. Injectability was determined according to (Equation 1):


(1) $$\;\text{Injectability}\;\left(\%\right)\;=\;{\text{W}}_{\text{e}}/{\text{W}}_{\text{i}}\times \;100\%$$

where W_e_ and W_i_ refer to the mass of extruded paste and the original mass of paste in the syringe, respectively.

For the cohesion assessment, the paste was extruded into 37°C PBS. Immediately after extrusion, the degrees of particulate cloud formation and fragmentation were qualitatively analyzed, and a score was given based on **Supplementary Table S1**. The cohesion score was calculated according to (Equation 2). Each assessment included three replicates per group (n = 3).


(2) $$\begin{array}{c} \text{Cohesion}\;\text{Score}\\ =\;\left(\text{particulate}\;\text{formation}\;\text{score}\;+\;\text{fragmentation}\;\text{score}\right)/2 \end{array}$$

### 2.4 Cell culture

Human osteosarcoma (Saos-2) and mouse pre-osteoblast (MC3T3) cells were obtained from the National Collection of Authenticated Cell Cultures, China. Saos-2 cells were cultured in McCoy’s 5A medium, supplemented with 15% fetal bovine serum (FBS), 100 units/mL penicillin, and 100 µg/mL streptomycin. MC3T3 cells were maintained in α-MEM, enriched with 10% FBS, 100 units/mL penicillin, and 100 µg/mL streptomycin. Both cell lines were incubated at 37°C in a humidified atmosphere with 5% CO_2_. Media were refreshed every 2-3 days, and cells were passaged at 80% confluence using 0.25% trypsin-EDTA.

### 2.5 Extract culture medium preparation

We employed the extract method for subsequent analyses. CS5P scaffolds, both with and without Cur, were hydrated for 3 days, immersed in 10 mL of either McCoy’s 5A medium (Saos-2) or α-MEM (MC3T3), and incubated at 37°C for 3 days to produce scaffold extracts. These extracts were then sterilized through a 0.22 µm filter. The resultant extracts were blended with McCoy’s 5A medium (for Saos-2, supplemented with 15% FBS) or α-MEM (for MC3T3, supplemented with 10% FBS), along with penicillin-streptomycin, to create the extract culture media. Control groups for Saos-2 and MC3T3 were the cells cultured in extract-free McCoy’s 5A medium and α-MEM, respectively.

### 2.6 CCK-8 assay

For the Saos-2 CCK-8 assay, 1×10^5^ cells in 200 μL of McCoy’s 5A medium were seeded per well in a 96-well plate and incubated overnight at 37°C with 5% CO_2_. The medium was then replaced with the extract culture medium for 1, 2, 3, and 4-day incubations before the CCK-8 assay. Optical density (OD) was measured at 450 nm using a microplate reader (CMax Plus, Molecular, U.S.A.), and the relative cell viability was calculated as [(OD_sample_ – OD_blank_)/(OD_control_ - OD_blank_)] × 100%. The MC3T3 CCK-8 assay followed the same protocol, with adjustments made only for the culture medium. Each group had three biological and five technical replicates (n = 15).

### 2.7 Flow cytometry

Cell apoptosis was assessed via flow cytometry. Briefly, 1×10^6^ cells were seeded per well in a 6-well plate and incubated overnight at 37°C with 5% CO_2_. After replacing the medium with the extract culture medium and a further 1-day incubation, the cells were detached using EDTA-free trypsin, stained following the Annexin V-APC/PI kit instructions, and analyzed using a flow cytometer (Accuri C6, BD Biosciences, U.S.A.).

### 2.8 ALP assay

The expression of ALP in MC3T3 cells cultured in the extract culture medium was assessed at 7 days. For the ALP assay, 5 × 10^5^ cells were seeded in each well of a 6-well plate, and 2 mL of 10% FBS-supplemented α-MEM culture medium was added per well. After 1 day of culture, the plate was rinsed with PBS, and the extract culture medium was added. After another 3 days of culturing at 37°C with 5% CO_2_, the plate was rinsed with PBS twice, and the ALP activity of each well was determined using an ALP assay kit. Each group had three biological replicates (n = 3).

### 2.9 Statistical analysis

In this study, the statistical analysis was performed using OriginPro (Education edition, OriginLab, U.S.A). One-way ANOVA was used to determine the statistical significance among groups, and a *p*-value < 0.05 (*) was considered statistically significant. Results are presented as mean ± standard deviation.

## 3 Results and discussion

**Figure 1:**
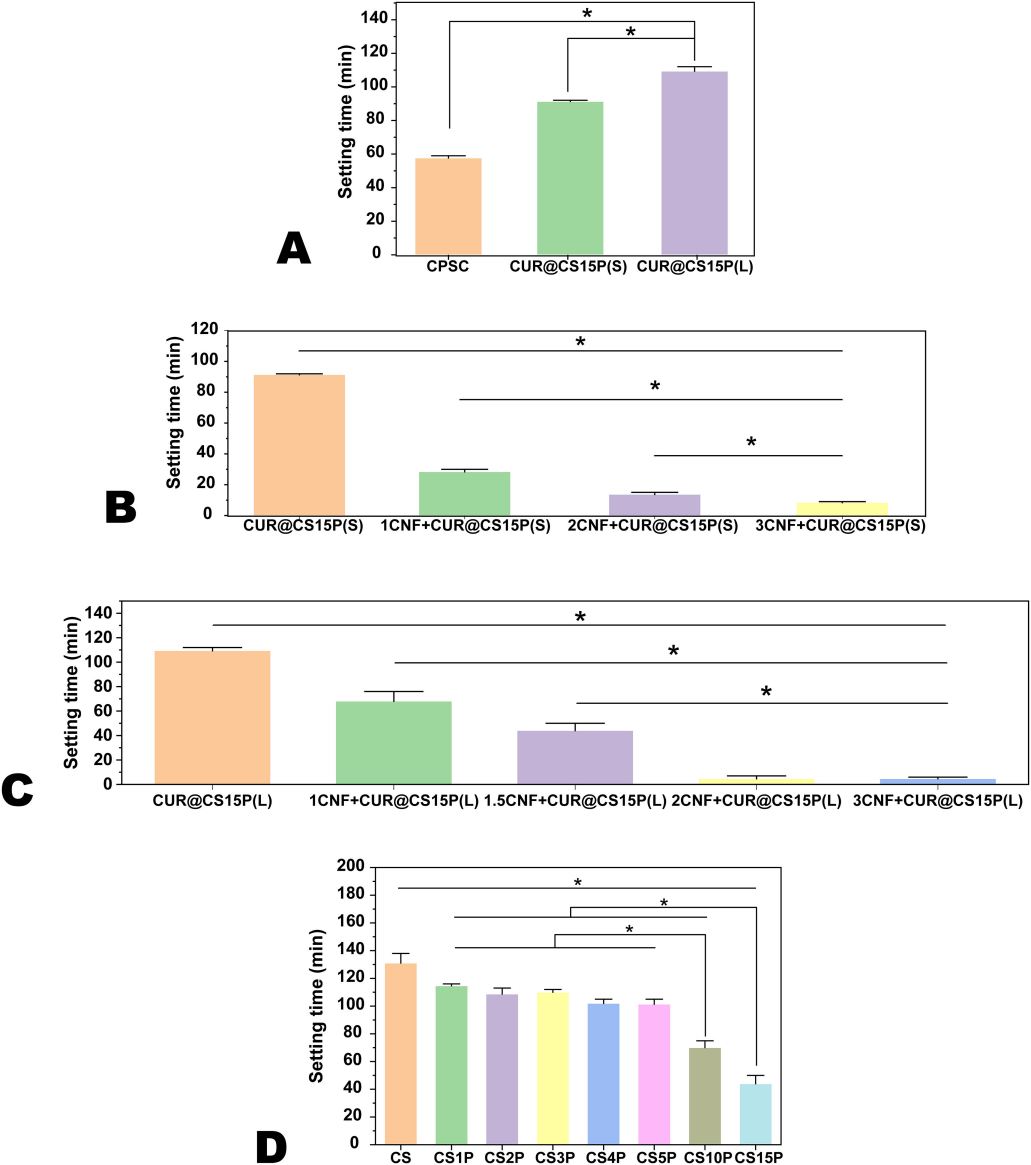
The setting time of Cur-CPSC. **(A)** The impacts of adding Cur strategy ( liquid or solid) on the setting time of CPSC with 5% mass fraction of Cur, **(B)** the impacts of CNF-SH on the setting time of CPSC with 5% mass fraction of Cur added in the solid form, **(C)** the impacts of CNF-SH on the setting time of CPSC with 5% mass fraction of Cur added in the liquid form, and **(D)** the impacts of MCP on the setting time of CPSC with 5% mass fraction of Cur added in the liquid form and 1.5% mass fraction of CNF-SH. The results are presented as mean ± standard deviation (n = 3). * represents the significance level at p < 0.05.

In this study, our primary objective was to develop an injectable formulation that is easy to handle and can adapt to various geometries of bone defects following tumor tissue removal from the bone. We thus assessed the setting time performance with and without the addition of curcumin (Cur). Given Cur’s water solubility, we prepared two formulations. In the first formulation (CUR@CS15P(S)), Cur was mixed with the CPSC, followed by hydration with deionized water (ddH_2_O). As depicted in **Figure 1A**, this approach markedly prolonged the setting time compared to the Cur-free CPSC, shifting from 57.67 ± 1.15 min to 91.33 ± 0.58 min. Conversely, pre-dissolving Cur in ddH_2_O before hydrating the CPSC led to an even greater increase in setting time to 109.33 ± 4.62 min. These findings suggest that the inclusion of Cur can significantly influence the hydration process of CPSC, likely due to Cur being adsorbed onto the unreacted calcium silicate (CS), thereby forming a barrier that inhibits further reaction with water (22). Although a prolonged setting time enhances the CPSC paste’s manageability, facilitating more precise application by medical professionals, it may compromise the paste’s stability within the bone defect, potentially leading to undesirable leakage (23).

Consequently, we explored modulating the setting time by incorporating silanized cellulose nanofiber (CNF-SH). Previous studies have shown that CNF-SH can significantly enhance the CPSC’s manageability and mechanical strength (21). In this study, we discovered that adding CNF-SH substantially shortened the setting time for both preparation methods (as illustrated in **Figures 1B, C**), agreeing with our previous findings. Since excessively brief setting times [e.g., 13.67 ± 1.15 min for 2CNF+CUR@CS15P(S) and 4.67 ± 2.08 min for 2CNF+CUR@CS15P(L)] can render a paste inapplicable, we determined that a 1.5% mass fraction of CNF-SH in CPSC yields an average setting time of 44 ± 5.29 min for 1.5CNF+CUR@CS15P(L). Moreover, because pre-dissolving Cur in water before mixing with CPSC was found to easier to prepare a more uniform paste than mixing Cur with CPSC in the powder state, we opted for this CNF-SH mass fraction for further studies.

**Figure 2:**
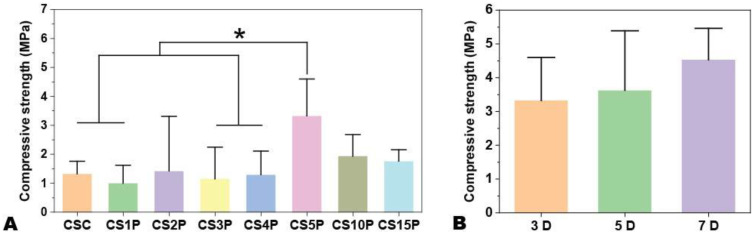
The compressive strength of Cur-CPSC samples with 1.5% CNF-SH and 5% Cur (L). **(A)** The impacts of MCP mass fraction on the Cur-CPSC's compressive strength, and **(B)** The impacts of hydration duration on the Cur-CPSC's compressive strength. The results are presented as mean ± standard deviation (n = 3). * represents the significance level at p < 0.05.

Beyond CNF-SH, monocalcium phosphate (MCP) emerged as another critical factor influencing both the setting time and mechanical strength of CPSC. We delved into the impact of MCP on setting time, as illustrated in **Figure 1D**. The hydration of CPS involves two steps. First, C_2_S and C_3_S react with ddH_2_O to form calcium silicate hydrate (CSH) and Ca(OH)_2_. In the second step, MCP further reacts with Ca(OH)_2_ to form apatite. According to Le Chatelier’s principle, adding MCP is expected to shift the reaction in favor of faster CSH formation, thereby reducing the setting time. Consistent with prior observations, the inclusion of MCP markedly reduced the setting time, shifting from 131 ± 6.56 min in MCP-free CS to 44 ± 5.29 min in samples with a 15% MCP mass fraction (19). Notably, at lower MCP concentrations (1% to 5%), the setting time remained relatively stable (**Figure 2A**).

The compressive strength of the scaffolds, evaluated after 3 days of hydration (**Figure 2B**), revealed that samples with a 5% MCP mass fraction exhibited the highest compressive strength at fracture (e.g., 3.33 ± 1.08 MPa for CS5P compared to 1.77 ± 0.27 MPa for CS15P). Furthermore, we investigated the influence of hydration duration on the mechanical strength of CPSC using the CS5P group. **Figure 2B** demonstrates that while strength tended to increase from 3.33 ± 1.08 MPa after 3 days to 4.54 ± 0.96 MPa after 7 days of hydration, the difference was not statistically significant, indicating a minimal effect of hydration duration on the mechanical properties of this Cur-incorporated CPSC formulation.

**Figure 3:**
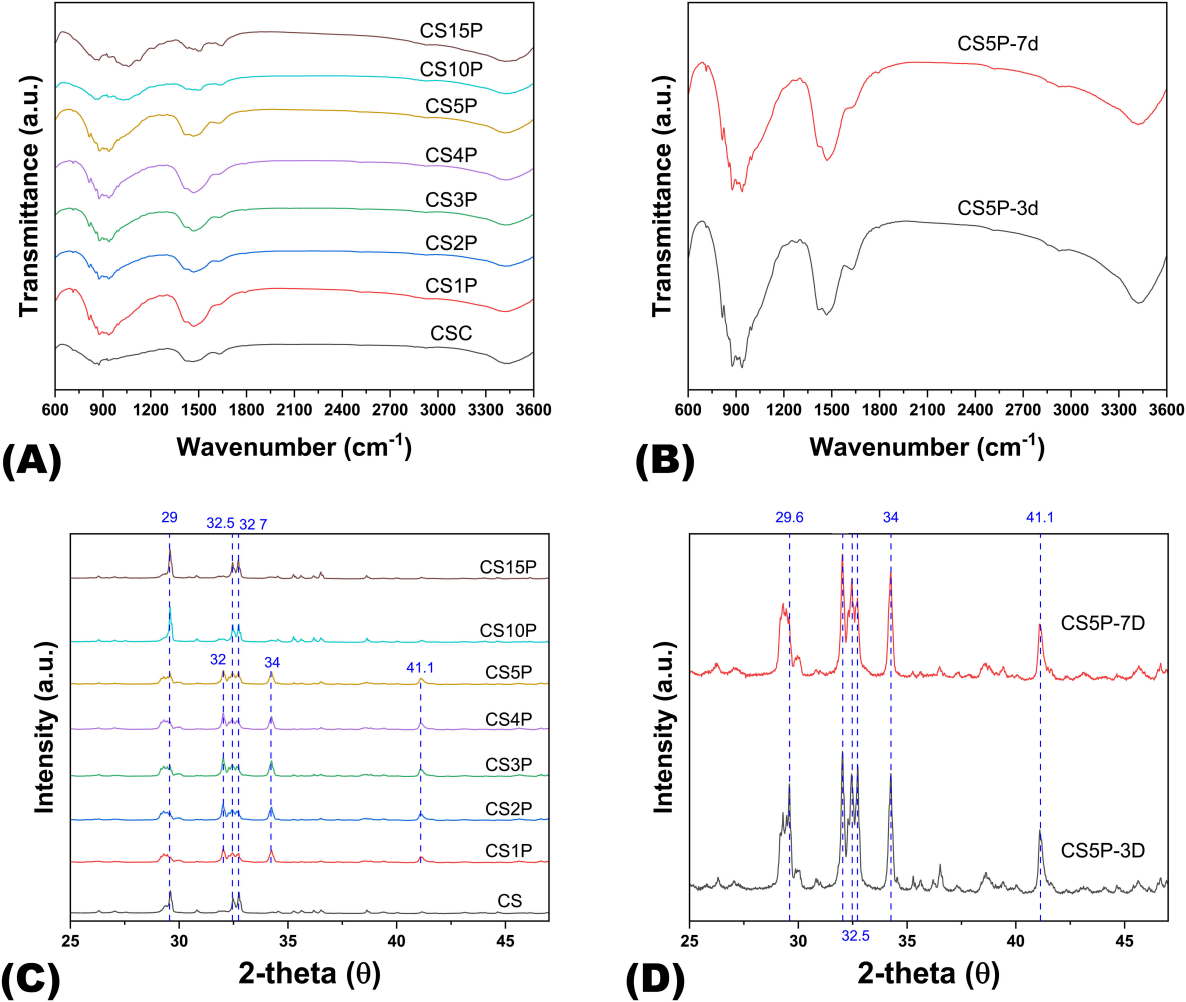
The FTIR spectra and XRD patterns of Cur-CPSC samples. **(A)** The impacts of MCP on the Cur-CPSC spectra, and **(B)** The impacts of hydration duration (3 and 7 days) on the spectra of Cur-CPSC containing 5% MCP, 5% Cur and 1.5% CNF-SH. **(C)** The impacts of MCP on the Cur-CPSC's XRD patterns, and **(D)** The impacts of hydration duration (3 and 7 days) on the XRD patterns of Cur-CPSC containing 5% MCP, 5% Cur and 1.5% CNF-SH.

To elucidate the impact of varying MCP quantities and hydration durations on Cur-CPSC formulations, we conducted XRD and FTIR analyses to uncover the underlying mechanisms. The FTIR spectra of samples with differing MCP mass fractions (**Figures 3A, B**) revealed the characteristic phosphate (PO_4_^3−^) absorption band around 1040 cm^−1^. The band around 860 cm^−1^ was attributed to the P-OH stretching vibration in HPO_4_^2−^. Furthermore, the overlapping absorption bands near 950 cm^−1^ corresponded to P-O and Si–O stretching, while the bands at 1430 cm^−1^ were linked to the combined effects of C-H bending and the stretching vibrations of CH_2_ and COO^−^ groups. Moreover, the band at 1640 cm^−1^ represented the symmetric stretching vibration of the COO^−^ group (24).

In the XRD analysis (**Figures 3C, D**), peaks at 29° were associated with unreacted dicalcium silicate (C_2_S) and the formation of calcium carbonate (CaCO_3_). Multiple peaks around 32° also indicated unreacted C_2_S, while peaks near 34° were assigned to calcium silicate hydrate (CSH) gel. In addition, peaks at 41° suggested incomplete hydration of tricalcium silicate (C_3_S). The XRD patterns clearly showed that at lower MCP mass fractions, the formation of CSH gel was evident, suggesting the hydration of CPSC occurs in two steps: initial reaction of C_2_S and C_3_S with water to form Ca(OH)_2_ and amorphous CSH, which eventually crystallizes into CSH gel, imparting mechanical strength to the hardened CPSC (25). The slower hydration rate of C_2_S, compared to C_3_S, accounted for the residual C_2_S observed in all samples. Subsequently, Ca(OH)_2_ may react further with MCP to form apatite or with dissolved CO_2_ to form calcium carbonate.

Increasing the MCP mass fraction from 0% to 5% favored the hydration of CS due to MCP’s consumption of Ca(OH)_2_, promoting CSH formation and, consequently, enhancing mechanical strength in correlation with MCP concentration. However, at higher MCP mass fractions (10% and 15%), excessive Ca(OH)_2_ consumption is encountered, diminishing the alkalinity of the Cur-dissolved water. Since Cur dissolution is optimized in an alkaline environment, a reduction in pH leads to decreased Cur solubility, resulting in Cur precipitation and adsorption onto amorphous CSH (26). This, in turn, affects the polymerization of CSH into CSH gel, diminishing the material’s mechanical properties.

**Figure 4:**
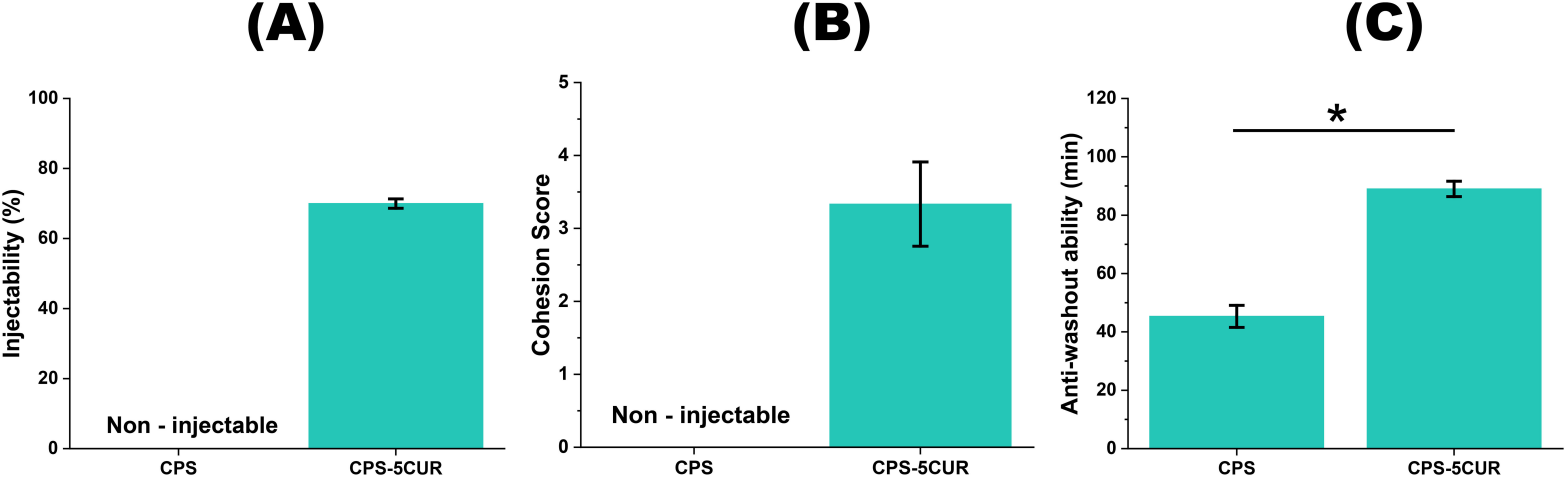
The injectability **(A)**, cohesion **(B)** and anti-washout ability **(C)** between CPS-5CUR and Cur-free CPS formulations. * represents the significance level at p < 0.05.

Considering these mechanical and chemical interactions, we ultimately selected a CPSC formulation with 5% MCP, 1.5% CNF-SH, and 5% Cur (referred to as CPS-5CUR in the following analyses) for subsequent experiments. Additionally, we evaluated this formulation’s injectability, cohesion, and anti-washout ability, which are crucial characteristics of an injectable self-setting bone substitute. As shown in **Figure 4**, compared to the Cur-free formulation, the CPS-5CUR formulation was found to be injectable using a syringe and exhibited acceptable anti-washout ability.

In the current investigation, our primary ambition was to pioneer the development of an injectable bioceramic bone substitute formulation that not only exhibits potent anti-tumor properties but also simultaneously fosters bone growth, thereby offering a dual-functionality crucial for post-surgical bone defect treatments following tumor excisions. Building upon our extensive groundwork in previous studies, where the promising capabilities of calcium phosphate silicate cement (CPSC) in bone regeneration were meticulously examined, we ventured to incorporate curcumin (Cur), a naturally occurring compound known for its mild antitumor activity, into the CPSC matrix (27). This strategic choice was motivated by the intention to circumvent the adverse toxicological effects commonly associated with more potent antitumor agents such as 5-Fluorouracil (5-FU), which, despite their efficacy, pose significant risks to the surrounding healthy tissues, particularly impairing the critical functions of osteoblast cells in bone proliferation and differentiation.

To methodically assess the potential cytotoxic effects of our Cur-enhanced biomaterial, we employed the extract method, meticulously preparing extracts from Cur-integrated CPSC and subsequently exposing two distinct cell lines to these extracts: the Saos-2 osteosarcoma cells to evaluate the anti-tumor efficacy and the MC3T3 pre-osteoblast cells to assess biocompatibility and potential cytotoxicity toward healthy bone-forming cells.

**Figure 5:**
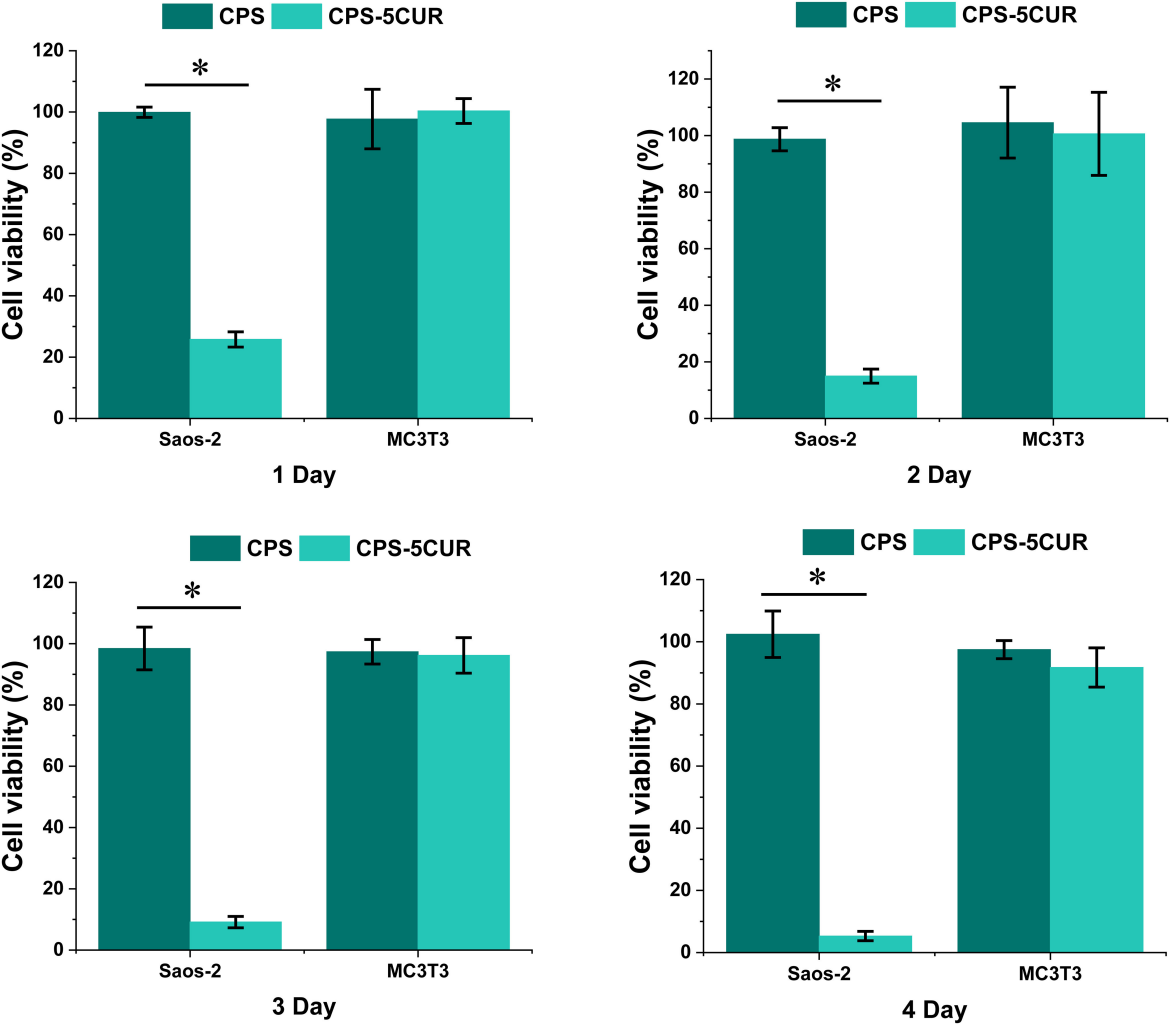
The cytotoxicity of osteosarcoma (Saos-2) and pre-osteoblast (MC3T3) cell lines incubated with Cur-CPSC extract culture medium from 1 day to 4 days. The results are presented as mean ± standard deviation (n = 15). * represents the significance level at p < 0.05. The cells treated with normal culture media served as the control groups.

Intriguingly, as depicted in **Figure 5**, our observations revealed a marked decrement in the viability of Saos-2 cells upon exposure to Cur-laden CPSC extracts across all evaluated time points (from 25.78% ± 2.50% in the 1^st^ day to 5.3% ± 1.51% in the 4^th^ day), underscoring the potentiated antitumor activity imparted by Cur. Furthermore, a noteworthy trend emerged, illustrating a time-dependent amplification of Cur’s antitumor effects, with prolonged exposure correlating with more pronounced reductions in cell viability. This temporal dependency aligns with the physiological timeline of bone remodeling, which spans approximately 1 to 3 months, suggesting that the prolonged degradation profile of CPSC, extending beyond a month, can facilitate sustained Cur release, thereby ensuring an enduring therapeutic action against residual osteosarcoma cells and potentially other malignancies within bone tissues (28). Moreover, the consistent viability observed in Saos-2 cells treated with Cur-free CPSC samples serves as a testament to the inherent non-tumorigenic nature of the CPSC matrix, reinforcing its suitability as a bone substitute material.

Further extending our investigation to the realm of biocompatibility, we meticulously evaluated the impact of Cur-CPSC extracts on the viability of MC3T3 pre-osteoblast cells over a 4-day incubation period. Remarkably, our findings indicated no significant alterations in cell viability attributable to Cur treatment, irrespective of the exposure duration (from 100.34% ± 4.1% in the 1^st^ day to 91.73% ± 6.32% in the 4^th^ day). This observation not only reaffirms the biocompatibility of the Cur-CPSC composite but also highlights its potential to support healthy bone tissue growth without impeding the vital processes of pre-osteoblast proliferation and differentiation (16, 29).

**Figure 6:**
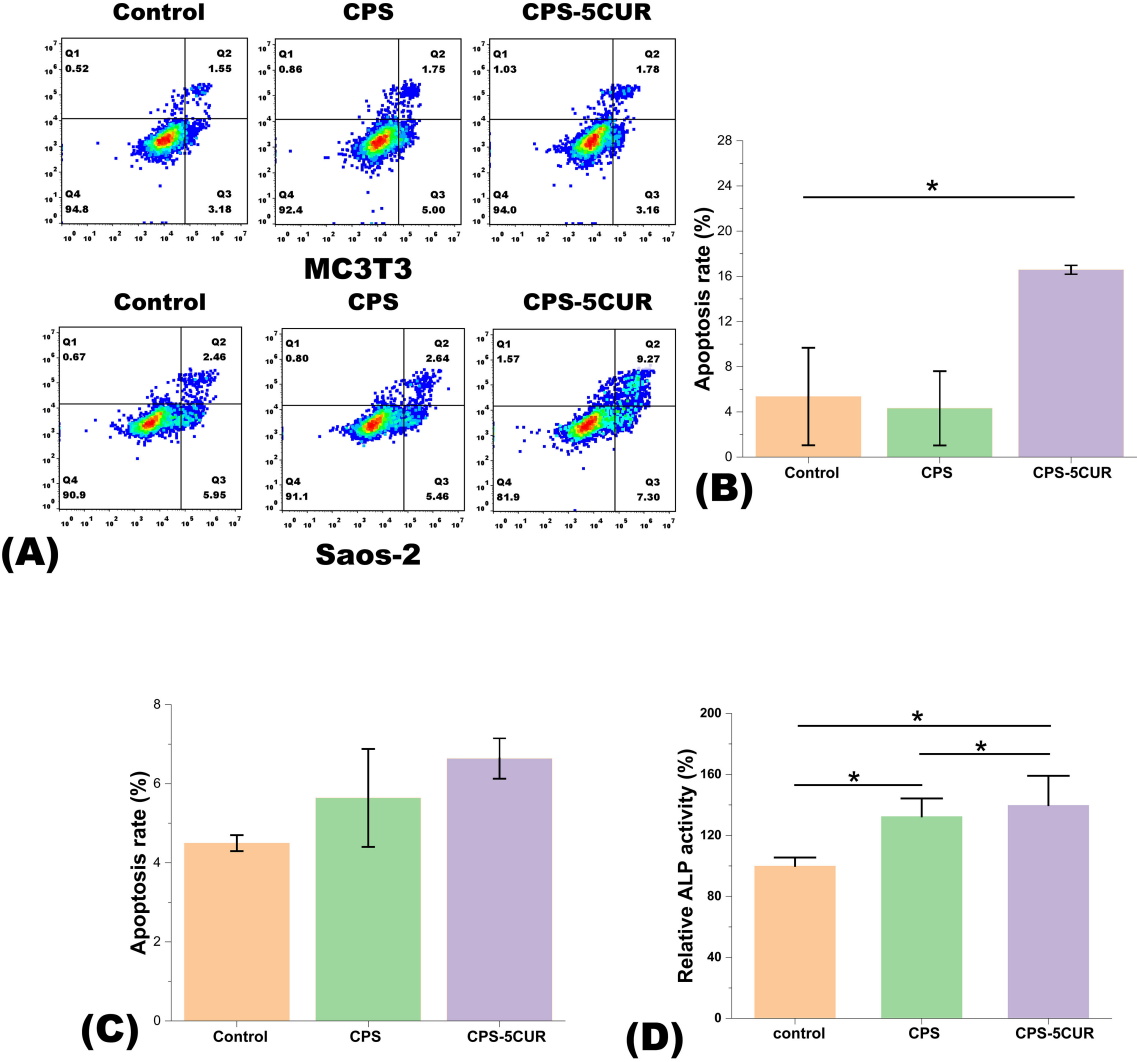
**(A)** Apoptosis was analyzed in both Saos-2 and MC3T3 cell lines treated with control, CPSC and Cur-CPSC for 1 day using flow cytometry. **(B)** The apoptosis rate (%) for the Saos-2 cell line and **(C)** the apoptosis rate (%) for the MC3T3 cell line. The apoptosis rate equals the sum of the proportions in Q2 and Q3. **(D)** The ALP activity of MC3T3 cells cultured with CPS-5CUR and CPS for 7 days. The results are presented as mean ± standard deviation (n = 3). * represents the significance level at p < 0.05.

To substantiate these findings, we conducted flow cytometry analyses, as detailed in **Figure 6**, which further elucidated the differential responses elicited by Cur treatment. A significant upsurge in apoptosis was observed in Saos-2 cells following a 1-day incubation with Cur-containing extracts, a clear indicator of Cur’s targeted antitumor activity. Conversely, the apoptosis rates in MC3T3 cells remained negligible under similar treatment conditions, thereby reinforcing the selective cytotoxic profile of Cur against tumor cells, while sparing healthy osteoblasts. Moreover, we assessed the production of ALP in osteoblast cells cultured with CPS-5CUR for 7 days. As shown in **Figure 6D**, ALP production in the CPS-5CUR and CPS groups significantly increased compared to the control, suggesting that the released calcium, silicate, and phosphate ions improved osteoblast cell proliferation and differentiation. Additionally, the inclusion of CUR provided further benefits in promoting cell proliferation and differentiation (30).

Gomma studied Chemo-preventive efficacy of herbal extracts of curcumin, ginger, cloves and amygdaline, and analyzed *in vivo* by examination of the apoptosis rate of EAC tumor cells by flow cytometry. The studied found the herbal extracts of curcumin, ginger, cloves and amygdaline may have anti-tumoral immunity and anti-cancer potency and potential to reduce the resistance to cancer conventional chemotherapy and exert cancer chemo-protective approaches with low adverse effects (31). Previous research has demonstrated that curcumin, varying doses ranging from 12.5 to 25 μM of trigger apoptosis in osteoblasts through the generation of reactive oxygen species (ROS), the activation of JNK signaling, and subsequent induction of mitochondrial membrane depolarization and caspase activation (32). Numerous studies also have shown that curcumin delays the initiation and progression of NSCLC by affecting a wide range of molecular targets and cell signaling pathways including NF-kB, Akt, MAPKS, BCL-2, ROS and microRNAs (miRNAs) (33, 34).

In summary, the results of our present study compellingly advocate for the incorporation of Cur into CPSC as a highly promising strategy for the treatment of bone defects subsequent to the surgical excision of bone tumors. The specificity of Cur’s cytotoxic effects toward tumor cell lines, sparing healthy cells, positions this novel formulation as an advantageous candidate for clinical application. However, it is noteworthy that, despite existing literature suggesting Cur’s potential to enhance osteoblast proliferation and differentiation, our study did not conclusively demonstrate this effect, possibly due to the limited duration of our observational period, which may not have been sufficient to capture the full spectrum of Cur’s influence on osteoblast behavior. Additionally, the lack of suitable *in vivo* models for osteosarcoma in our study constrained our ability to thoroughly investigate the dual anti-tumor and bone-regenerative capacities of Cur-CPSC. Consequently, future studies, particularly those extending into *in vivo* domains, are warranted to further elucidate and validate the multifaceted therapeutic potential of Cur-enhanced CPSC in bone cancer treatment paradigms.

## 4 Conclusion

In this study, we developed an injectable curcumin (Cur)-infused calcium phosphate silicate cement (CPSC) as a potential bone substitute for repairing defects associated with bone cancer. Our analysis focused on the effects of Cur incorporation on CPSC’s workability, setting time, and mechanical strength, revealing that Cur significantly prolonged the setting time. To counter this, we enhanced the formulation by adding silanized cellulose nanofiber (CNF-SH), achieving an optimal balance of setting duration and mechanical robustness. Biocompatibility assessments indicated that Cur-CPSC was highly toxic to osteosarcoma cells while showing minimal cytotoxicity to pre-osteoblast cells. These findings suggest that Cur-CPSC holds promise as a candidate for bone defect repair following cancer removal, meriting further investigation.

## Data Availability

The original contributions presented in the study are included in the article/**Supplementary Material**. Further inquiries can be directed to the corresponding author.
