# Supplementary material for: Cellulose nanofiber reinforced curcumin-infused calcium phosphate silicate cement for various bone-tissue engineering application
Source: Front Oncol. 2025 Jan 14;14:1516638. doi: 10.3389/fonc.2024.1516638 (PMC11772290; doi:10.3389/fonc.2024.1516638)
Supplement: Supplementary file 1 [file Table1.docx]

| **Score** | **Particulate formation** | **Fragmentation** |
| --- | --- | --- |
| 5 | Virtually no particulate | A continuous paste |
| 4 | A few particulates observed | Paste length greater than 5 cm |
| 3 | Particulate clouds formed visibly | Paste length between 1 and 5 cm |
| 2 | Large amounts of particulate clouds formed | Paste length shorter than 1 cm |
| 1 | Completely disintegrated | A discontinuous paste |

Table S1: The grading scheme to assign scores for the particulate formation and fragmentation in the cohesion study
